# Enhancing career adaptability and career decision-making self-efficacy through career planning education: a quasi-experimental study

**DOI:** 10.3389/fpsyg.2025.1612768

**Published:** 2025-12-17

**Authors:** Jingwen Zhang, Mansor Abu Talib, Jiajian Wang

**Affiliations:** 1 Faculty of Innovation and Entrepreneurship, Jilin Medical University, Jilin City, China; 2 Faculty of Social Sciences and Liberal Arts, UCSI University, Kuala Lumpur, Malaysia; 3 Wellbeing Research Centre, UCSI University, Kuala Lumpur, Malaysia; 4 Student Affairs Center, Sanming University, Sanming, China

**Keywords:** quality education, career planning education, career adaptability, career decision-making self-efficacy, quasi-experimental, undergraduates

## Abstract

**Introduction:**

Career adaptability (CA) and career decision-making self-efficacy (CDMSE) are key competencies for university students. This study examined the effectiveness of a structured, theory-based career planning education (CPE) program in enhancing these outcomes.

**Methods:**

Using a quasi-experimental design, 75 undergraduates were assigned to an experimental or control group. The five-week CPE intervention was grounded in career construction and social cognitive theories. Measures of CA and CDMSE were collected at pretest, posttest, and a four-week follow-up.

**Results:**

Results indicated that the experimental group showed significant improvements in CA and CDMSE at posttest. No significant changes were observed in the control group.

**Discussion:**

The findings support the short-term effectiveness of structured CPE programs, while highlighting the need for future research on long-term outcomes and broader institutional implementation.

## 1 Introduction

University students today are navigating an increasingly complex and uncertain world of work (Bal and Arikan, 2020). In this volatile, uncertain, complex, and ambiguous (VUCA) environment, the ability to adapt has become a critical competency for successful career development (Savickas et al., 2009). Among the various transitions students face, the move from school to work is particularly stressful and often accompanied by uncertainty, anxiety, and self-doubt (Kepir Sávoly and Tuzgol Dost, 2020). For medical and allied health undergraduates in China, the school-to-work transition is further complicated by stressful residency and employment competition, evolving health-care service demands and strong academic (Li et al., 2025; Mao et al., 2019; Yi et al., 2022).

Career adaptability (CA) has emerged as a key construct in addressing these challenges. Savickas (2005), within the framework of Career Construction Theory, defines career adaptability as “a psychological construct that implies an individual’s readiness and resources to cope with current and foreseeable career development demands, occupational changes, and personal traumas” (p. 45). Individuals with higher levels of CA tend to exhibit greater flexibility and resilience in coping with changing career demands (Savickas, 2013). As such, CA is closely associated with reduced stress and improved well-being in both academic and professional settings (Chui et al., 2022). In China, the school-to-work transition can be particularly daunting due to structural and cultural factors (Guan et al., 2016). While career exploration is widely encouraged, it does not always lead to greater decision-making readiness. Studies suggest that Chinese undergraduates often experience information overload or inconsistency during self-exploration, which may, paradoxically, hinder the development of career adaptability (Gu et al., 2020; Wang and Chang, 2025). Moreover, insufficient adaptability has been linked to increased risks of anxiety and depression (Shulman et al., 2014), further underscoring the importance of supporting students in developing this capacity. Recent longitudinal evidence indicates that CA support academic adjustment and achievement, implying that strengthening CA via career planning education (CPE) may also bolster academic efficacy and persistence (Yi et al., 2025).

In addition to career adaptability, career decision-making self-efficacy (CDMSE) represents a critical psychological resource in shaping individuals’ career development. The concept of CDMSE derives from Bandura’s (1977) Social Cognitive Theory and refers to an individual’s belief in their ability to successfully perform the tasks necessary for making effective career decisions, including evaluating options, aligning choices with interests and goals, and addressing potential barriers (Taylor and Betz, 1983; Betz et al., 1996). CDMSE plays a central role in career-related behaviors, influencing career exploration, goal setting, and persistence in the face of uncertainty (Penn and Lent, 2019). Individuals with high CDMSE are more likely to take initiative in navigating career pathways, adapt their plans as needed, and remain engaged throughout the decision-making process. Given its strong predictive value, CDMSE has become a central focus in the design of career interventions. Targeted programs that aim to enhance students’ decision-making efficacy have been shown to reduce indecision and facilitate smoother school-to-work transitions (Gu et al., 2020). Recent reviews indicate that CDMSE is positively associated with academic achievement and persistence (Liu et al., 2024).

Career planning interventions grounded in Career Construction Theory have demonstrated consistent effectiveness in enhancing students’ career adaptability (CA). Teychenne et al. (2019) found that an online module tailored to students’ interests improved adaptability across key dimensions. Similarly, Kepir Sávoly and Tuzgol Dost (2020) reported sustained gains in CA following a transition program, with effects lasting beyond the intervention. Bal and Arikan (2020) confirmed the value of structured classroom-based curricula, while Hur et al. (2018) showed that a coaching-based program reduced indecision and strengthened career readiness in medical students. In China, Gai et al. (2022) implemented a motivational interviewing approach among low-CA undergraduates and observed significant improvements in career control and confidence. Extending this work, Kim (2022) developed a 13-week online career adaptability improvement program embedded in the undergraduate curriculum, students who completed the program showed greater gains in overall CA than peers in standard career education courses. Wetstone and Rice (2023) reported that a brief, group-based intervention for university students also produced significant improvements in career adaptability relative to a comparison group. Life-design interventions grounded in Career Construction Theory show similar patterns: Cardoso et al. (2022), for example, found that a group life-design program with adolescents enhanced vocational identity, CA and CDMSE at post-test and three-month follow-up. Synthesizing these and other trials, Soares et al.’s (2022) systematic review of career interventions for university students concluded that theory-based, group-format programs generally yield positive changes in decision-making skills and adaptability-related resources. Taken together, these findings highlight the potential of CPE to strengthen adaptability-related resources.

A growing body of research highlights the effectiveness of career education programs in enhancing career decision-making self-efficacy (CDMSE). Interventions grounded in Social Cognitive Theory (Talib et al., 2015; Osborn et al., 2020) have demonstrated measurable gains in students’ confidence to navigate career decisions. Lam and Santos (2018) employed a multi-modal intervention combining lectures, assignments, and personal reflections, which led to reduced career indecision and enhanced CDMSE among Malaysian undergraduates. Other studies have validated the sustained impact of career interventions across diverse cultural and disciplinary settings, including motivational and counseling-based programs for undergraduates in Nigeria (Chukwuedo et al., 2021) and Indonesia (Putri et al., 2019). Several of these interventions have also been adapted to support medical students’ career development and readiness for professional transitions (EI Naggar et al., 2021). A 2024 review (Flint, 2024) of 15 peer-reviewed studies concluded that university career development interventions, courses, workshops, and online modules, consistently raise CDMSE, especially when grounded in self-efficacy and built around structured practice and feedback. Meta-analytic evidence also points to robust effects: Özlem (2019) found that career interventions for university students were associated with a large overall increase in CDMSE, and Sharapova et al.’s (2023) review of school-based career guidance reported consistent benefits for students’ career-related beliefs, including decision-making self-efficacy. Taken together, these findings suggest that structured, theory-informed CPE delivered in group or course formats can reliably strengthen CDMSE alongside other career development outcomes.

Despite these encouraging findings, important gaps remain. First, most empirical work has treated CA and CDMSE as separate targets, designing interventions either to foster adaptability or to build decision-making confidence, rather than examining them as joint outcomes of a single, integrated CPE program. Second, existing evaluations have mainly involved general undergraduate samples. Much less is known about how theoretically informed CPE can strengthen CA and CDMSE among Chinese medical and allied health students, whose training routes and career pressures differ from those of other majors. Third, although Career Construction Theory and Social Cognitive Theory are often invoked as guiding frameworks, few studies have translated them into a clearly coordinated set of learning activities, for instance, linking narrative and future-oriented exercises to adaptability, while using structured practice, role modeling, and feedback within discipline-specific tasks to build decision-making self-efficacy.

While career education interventions have demonstrated effectiveness in improving career adaptability (CA) and career decision-making self-efficacy (CDMSE), evidence regarding whether their impact varies by gender remains mixed. Several studies have reported that female students tend to benefit more from career-related interventions.

This study aims to develop, validate and implement a CPE program for undergraduate students and to evaluate its effectiveness in enhancing students’ CA and CDMSE. The following hypotheses are proposed:

*H1:* There will be a meaningful short-term increase in CA and CDMSE in the experimental group at T2.

*H2:* There will be a significant improvement in both CA and CDMSE at follow-up (T3) relative to baseline (T1) in the experimental group.

*H3:* At post-test (T2) and follow-up (T3), students in the experimental group will score higher on CA and CDMSE than those in the control group.

## 2 Methods

### 2.1 Participants

This quasi-experimental study was conducted over a five-week period from September to October 2024 at Jilin Medical University, Jilin Province, China. Ethical approval was obtained from the Institutional Ethics Committee of UCSI University (Approval No. IEC-2024-FOSSLA-0052). Two intact classes were randomly selected to participate in the study. All students in each selected class were invited to participate, no individual-level randomization was performed. Students majoring in Health Services and Management were assigned to the experimental group, while those majoring in Medical Information Engineering formed the control group. The experimental group consisted of 40 students (12 males and 28 females), and the control group consisted of 35 students (14 males and 21 females). Gender composition did not differ between groups, *χ*^2^ (1, *N* = 75) = 2.47, *p* = 0.125.

### 2.2 Procedure

This study used a quasi-experimental design with a waiting-list control. Two intact classes were selected for participation, allocation to condition followed pre-existing class membership rather than individual randomization (Johnson and Christensen, 2000). To mitigate selection bias, baseline (T1) measures were collected before the intervention, and baseline equivalence at T1 was assessed on demographics and outcomes. The experimental group received the Career Planning Education (CPE) intervention, and the control group was scheduled to receive it after study completion. Outcomes were assessed at T1 (pretest), T2 (immediate post-intervention), and T3 (4-week follow-up) following Lam and Santos (2018). This multi-wave data collection allowed for the evaluation of both immediate and sustained effects of the intervention.

All questionnaires were administered online using Questionnaire Star.^1^ Prior to participation, students received detailed information about the study and provided written informed consent. Anonymity and confidentiality were strictly maintained throughout the research process.

Data was analyzed using SPSS 26. To evaluate Hypotheses, a multivariate analysis of variance (MANOVA) was conducted to assess group and time effects on CA and CDMSE.

### 2.3 The career planning education program

The Career Planning Education (CPE) program was developed based on Career Construction Theory (Savickas, 2005), Social Cognitive Theory (Bandura, 1977) and informed by a comprehensive literature review. The theoretical structure of the program centered on two core components. The first, career adaptability, draws from Savickas’s four-dimensional framework, including career concern (future orientation and planning), career control (self-directed action), career curiosity (exploration of options), and career confidence (belief in one’s capacity to solve problems and achieve goals). The second component, career decision-making self-efficacy, is rooted in Bandura’s theory and includes five task dimensions, self-appraisal, occupational information gathering, goal selection, planning, and problem solving (Betz et al., 1996). Instructional strategies were also designed to activate the four sources of self-efficacy, performance accomplishments, vicarious learning, verbal persuasion, and physiological/emotional regulation, to enhance students’ beliefs in their capacity to make effective career decisions.

Although the overall structure of the CPE module could be applied to other disciplines, the curriculum in this study was tailored to allied medical students. Teaching materials drew on clinical cases, such as doctor-patient communication, interprofessional teamwork and competency frameworks for health professionals. Group activities required students to examine typical career trajectories of allied health workers in China, to compare different specialties and practice settings, and to link their own plans to concrete stages of medical training such as internship. In doing so, the module integrated general career-planning skills with the exploration of professional identity in medicine.

To ensure content validity and contextual relevance, the draft program was reviewed by three experts in career education and psychology, whose feedback was incorporated prior to implementation.

To ensure instructional fidelity, lecturers received targeted training sessions covering the program’s theoretical foundations, research framework, and detailed lesson plans.

The primary objective of the program was to shift the focus of career education from traditional person-job matching to the active construction of a lifelong career path. The program’s objectives were to enhance students’ self-understanding (interests, abilities, and values), broaden their career awareness, strengthen decision-making skills, and support the formulation of both short- and long-term career goals.

The program adopted a group-based learning model. To promote active participation and reduce anxiety associated with group work, students were allowed to form self-selected groups of 4–6 members. This approach fostered a psychologically safe and emotionally supportive environment, aligning with Bandura’s concept of *physiological and emotional regulation* as a source of self-efficacy.

To support learning and reflection, students used a career development handbook to record their insights throughout the course. They were encouraged to share their reflections either within small groups or through class-wide presentations. This process represents *performance accomplishments*, the most powerful source of self-efficacy in Bandura’s framework, as it allows students to experience and demonstrate mastery.

Additionally, structured peer feedback was incorporated during group discussions, enabling students to observe others’ progress and receive constructive input. This reflects the mechanism of *vicarious learning*, where observing peer performance and receiving encouragement enhances students’ belief in their own abilities.

Throughout the sessions, instructors provided targeted verbal encouragement to affirm students’ efforts and progress, particularly during reflection sharing and group discussions. This strategy aligns with Bandura’s concept of *verbal persuasion*, which emphasizes the role of positive reinforcement in strengthening learners’ beliefs in their own capabilities.

The program spanned 5 weeks and consisted of two 1.5-h sessions per week, totaling 10 sessions in all. The full curriculum schedule is shown in Table 1.

**Table 1:** Teaching content of career planning education.

No.	Topic	CCT	SCT	CDMSE dimensions	Content	Activity
1	Building a medical career vision	Concern, Control Curiosity	Performance attainments, Vicarious experiences, Verbal persuasion, Emotional states		Career Transitions in Healthcare Systems Career Development Stage Theory Constructing a Career Vision	Interactive teamwork, Lecture support and feedback Discussion in teams, Presentation on preferred medical career paths
2–3	Career exploration and outlook in medicine	Concern, Control Curiosity	Performance attainments, Vicarious experiences, Verbal persuasion, Emotional states	Information Gathering, Preparation, Problem Solving	Expanding Career Vision in Healthcare Enriching Career Awareness Exploring the Full Scope of Careers	Independent research using health system information Interactive teamwork, Lecture support and feedback Discussion in teams, Presentation
4	Career interests in the medical field	Concern, Control Curiosity	Performance attainments, Vicarious experiences, Verbal persuasion, Emotional states	Self-Appraisal	Interest Holland’s theory Linking interests to Medical Specialties	Self-assessment, Discussion in teams Presentation on preferred specialties Lecture support and feedback
5	Career values in healthcare	Concern, Control Curiosity	Performance attainments, Vicarious experiences, Verbal persuasion, Emotional states	Self-Appraisal	Values, Professional Values in Medicine Clarify Your Values Values in Career Decision-Making	Self-assessment, Presentation Discussion in teams based on short clinical vignettes Lecture support and feedback
6–7	Career skills for future health professionals	Concern, Control Confidence	Performance attainments, Vicarious experiences, Verbal persuasion, Emotional states	Self-Appraisal, Goal Selection, Information Gathering	Employer Expectations for Health Workers Clarifying Personal Abilities Achievement Events	Self-assessment, Presentation Discussion in teams using clinic situations Lecture support and feedback
8	Career decision in medicine	Control	Performance attainments, Vicarious experiences, Verbal persuasion, Emotional states	Problem Solving	What is Career Decision-Making? Decision-Making Tools for Choosing Specialties	Peer assessments Presentation of individual decision cases Lecture support and feedback
9	Goal setting and medical career planning	Concern, Control Curiosity	Performance attainments, Vicarious experiences, Verbal persuasion, Emotional states	Goal Selection, Preparation	SMART Principle, Using the Fishbone Diagram Embracing Change in the Medical Labor Market	Individual consultation, Presentation, Lecture support and feedback
10	Medical career development report	Control, Confidence	Performance attainments, Vicarious experiences, Verbal persuasion, Emotional states	Self-Appraisal, Goal Selection, Preparation	Students’ Presentation	Individual consultation, Presentation, Lecture support and feedback

CCT, Career Construction Theory (Savickas, 2005); SCT, Social Cognitive Theory (Bandura, 1977, 1986).

### 2.4 Measures

All measures employed in this study were previously validated and demonstrated good reliability in Chinese samples. All items were rated on a 5-point Likert scale ranging from 1 (strongly disagree) to 5 (strongly agree).

#### 2.4.1 Career adapt-abilities scale-China form

Career Adapt-Abilities Scale-China Form (Hou et al., 2012) consists of four domains and 24 items, which is the Chinese version of Career Adapt-Abilities Scale-International Form 2.0 (Savickas and Porfeli, 2012). This questionnaire consists of four facets that measure different aspects of career, concern, control, confidence and curiosity. Example items are, thinking about what my future will be like (concern), counting on myself (control). Higher scores indicate better career adaptability.

#### 2.4.2 Career decision making self-efficacy scale-short form-China

The Chinese version of the Career Decision Making Self-Efficacy Scale-Short Form (Hampton, 2006) consists of five domains and 25-items, which was developed based on the English version of the CDMSE-SF (Betz et al., 1996). This scale measures five aspects of self-efficacy, including self-appraisal, gathering information, goal selection, planning and problem solving. Example items are, accurately assess your abilities (Self-appraisal), Find information about occupations you are interested in (Information gathering). Higher scores mean respondents have a high level of confidence to complete career related tasks.

### 2.5 Pilot testing

Both instruments were pilot tested with a randomly selected class of 41 students majoring in Health Inspection and Quarantine. The results demonstrated excellent internal consistency in the current context, with Cronbach’s *α* values of 0.98 for the Career Adapt-Abilities Scale-China Form (CAAS-CF) and 0.98 for the Career Decision-Making Self-Efficacy Scale-Short Form (CDMSE-SF). These findings provide a solid psychometric foundation for the main study.

## 3 Results

### 3.1 Preliminary analyses

Descriptive statistics for career adaptability (CA) and career decision-making self-efficacy (CDMSE) across the three time points and two groups are presented in Table 2. Normality was assessed using skewness and kurtosis values, which are commonly used indicators of distribution shape. According to Bulmer (1979), values between −1 and +1 suggest approximate normality. All variables at the three points met this criterion (see Table 2), supporting the use of parametric statistical analyses.

**Table 2:** Descriptive statistics.

Variable	Time point	Experimental				Control			
		*M*	SD	Skewness	Kurtosis	*M*	SD	Skewness	Kurtosis
CA	Pre	3.545	0.457	0.119	−0.319	3.567	0.548	0.142	0.345
	Post	4.007	0.365	−0.234	−0.173	3.588	0.504	−0.322	−0.344
	Follow-up	3.740	0.476	0.032	−0.523	3.653	0.634	0.742	−0.445
CDMSE	Pre	3.251	0.527	−0.037	0.141	3.469	0.638	0.024	0.348
	Post	3.814	0.504	0.030	−0.796	3.525	0.552	0.072	−0.455
	Follow-up	3.601	0.464	0.660	−0.217	3.589	0.613	0.517	0.038

CA, Career Adaptability; CDMSE, Career Decision-Making Self-Efficacy.

Independent samples *t*-tests were conducted to examine baseline equivalence. Results indicated no significant differences between the experimental and control groups on career adaptability (*t* = 0.190, *p* = 0.850) or career decision-making self-efficacy (*t* = 1.624, *p* = 0.109), suggesting group equivalence at baseline.

The assumption of homogeneity of variance was tested using Levene’s test, all *p*-values exceeded 0.05, supporting the assumption of equal variances.

A Pearson correlation analysis was conducted between career adaptability and career decision-making self-efficacy at the pretest (Time 1) across the full sample. The results indicated a significant positive correlation (*r* = 0.778, *p* < 0.01), suggesting that the two constructions are related, thereby supporting the use of Multivariate Analysis of Variance (MANOVA) to examine the effects of the intervention.

These findings justified the use of MANOVA to examine the effects of the career planning education program on both dependent variables simultaneously.

### 3.2 Effects of the intervention on CA and CDMSE

To examine the overall effect of the career planning education (CPE) program on career adaptability (CA) and career decision-making self-efficacy (CDMSE), MANOVA was conducted. The model included group (experimental vs. control) as the between-subjects factor and time (T1: pretest, T2: posttest, T3: follow-up) as the within-subjects factor. A MANOVA revealed a significant Group × Time interaction, Wilks’ Λ = 0.957, *F* (4, 436) = 2.304, *p* = 0.049, partial *η*^2^ = 0.022, indicating that changes in CA and CDMSE over time differed between the experimental and control group, with greater gains in the experimental class. Univariate tests are summarized in the next sections.

*H1: There will be a meaningful short-term increase in CA and CDMSE in the experimental group at T2.*

**Figure 1:**
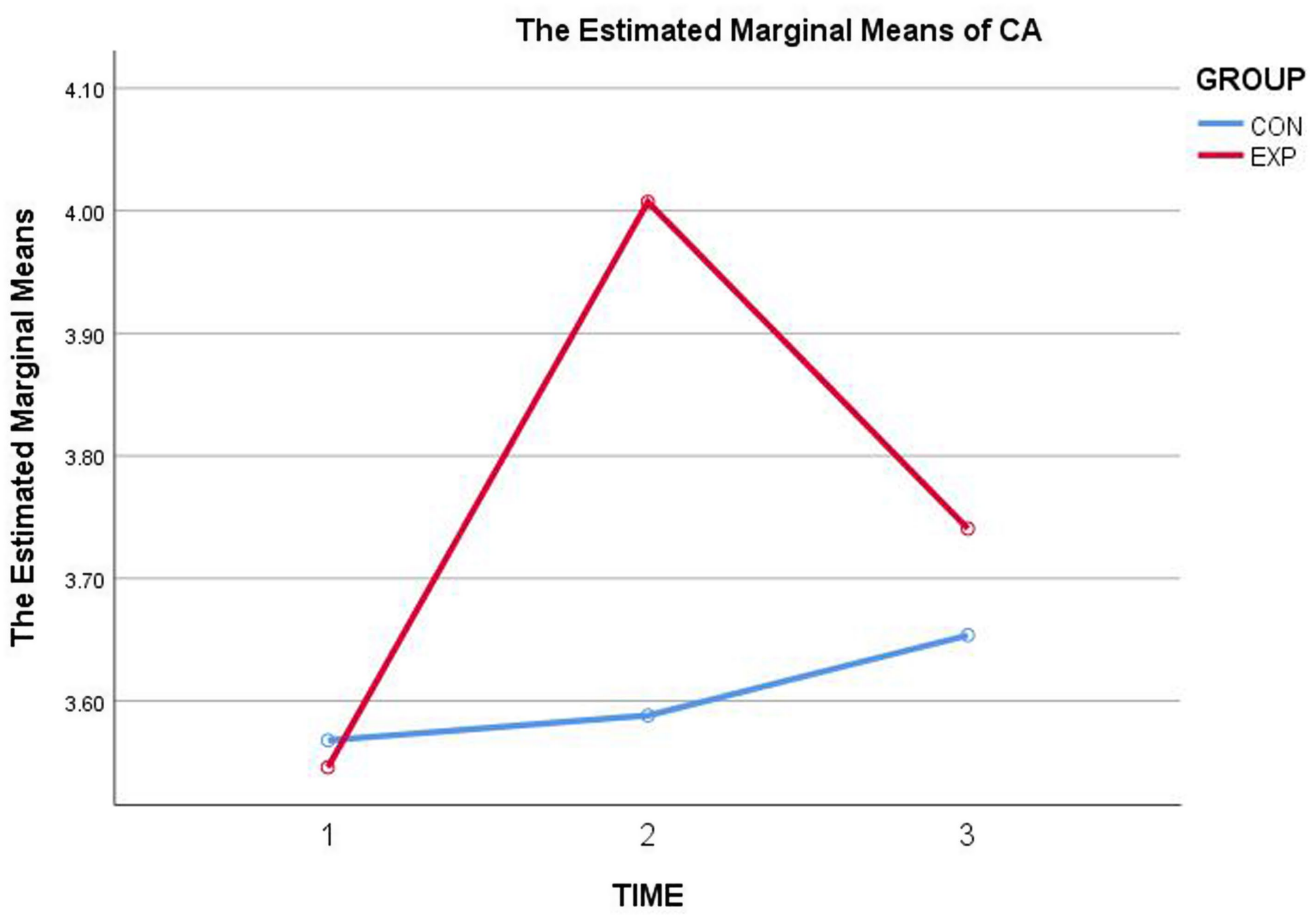
Estimated marginal means of CA across three time points for the experimental and control groups.

**Figure 2:**
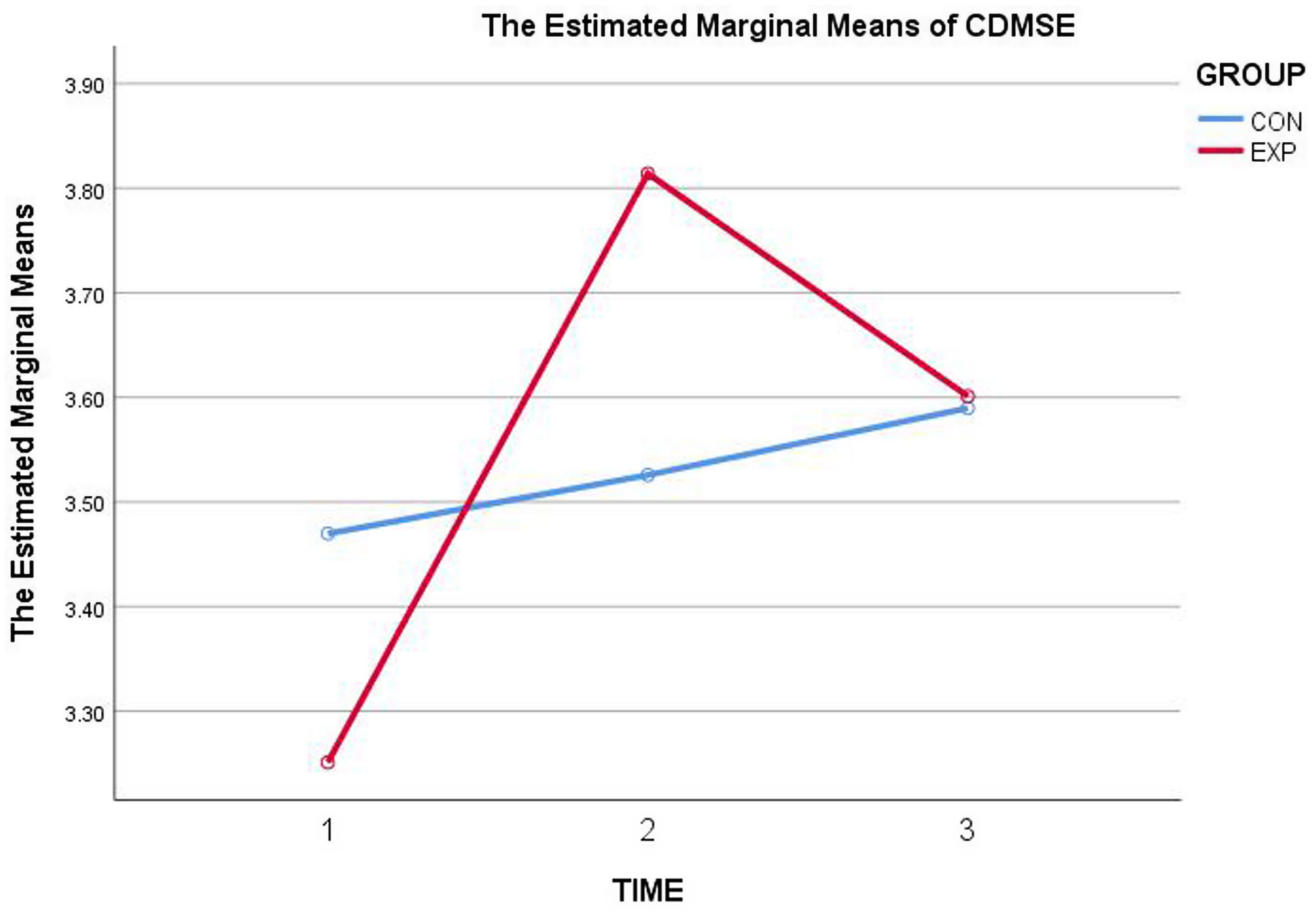
Estimated marginal means of CDMSE across three time points for the experimental and control groups.

CA increased from *M* = 3.545 (T1) to *M* = 4.007 (T2), *p* < 0.05 (Table 3, Figure 1), partial *η*^2^ = 0.072. CDMSE increased from *M* = 3.251 (T1) to *M* = 3.814 (T2), *p* < 0.05 (Table 3, Figure 2), partial *η*^2^ = 0.087. Thus, H1 was supported.

*H2: There will be a significant improvement in both CA and CDMSE at follow-up (T3) relative to baseline (T1) in the experimental group.*

CA. T3 (*M* = 3.740) was not significantly higher than T1 (*M* = 3.545), *p* > 0.05, partial *η*^2^ = 0.014 (Table 3; Figure 1), indicating attenuation from the posttest peak (T2 = 4.007) and no reliable maintenance above baseline. CDMSE. T3 (*M* = 3.601) remained significantly higher than T1 (*M* = 3.251), *p* < 0.05, partial *η*^2^ = 0.014 (Table 3; Figure 2), consistent with partial maintenance despite attenuation from T2 = 3.814. Thus, H2 was not supported as stated, although CDMSE showed partial maintenance above baseline whereas CA did not.

*H3: At post-test (T2) and follow-up (T3), students in the experimental group will score higher on CA and CDMSE than those in the control group.*

At T2, the experimental group exceeded the control group on CA (4.007 vs. 3.588), *p* < 0.05 (Table 4), partial *η*^2^ = 0.056. At T2, the experimental group exceeded the control group on CDMSE (3.814 vs. 3.525), *p* < 0.05 (Table 4), partial *η*^2^ = 0.023.

**Table 3:** Results of pairwise comparisons of within subjects.

Variable	Time (I)	Time (J)	EXP				CON			
			Mean difference (I-J)	SE	*p*	Partial *η*^2^	Mean difference (I-J)	SE	*p*	Partial *η*^2^
CA	2	1	0.461	0.112	0.000	0.072	0.020	0.120	0.998	0.000
	3	2	−0.267	0.112	0.053	0.025	0.065	0.120	0.928	0.001
	3	1	0.195	0.112	0.229	0.014	0.086	0.120	0.855	0.002
CDMSE	2	1	0.563	0.123	0.000	0.087	0.056	0.131	0.964	0.001
	3	2	−0.213	0.123	0.233	0.036	0.064	0.131	0.948	0.001
	3	1	0.350	0.123	0.014	0.014	0.120	0.131	0.741	0.004

**Table 4:** Results of pairwise comparisons of between-subject.

Variable	Time	GROUP (EXP-CON)	SE	*p*	Partial *η*^2^
CA	2	0.419	0.116	0.000	0.056
	3	0.087	0.116	0.453	0.003
CDMSE	2	0.288	0.127	0.024	0.023
	3	0.011	0.127	0.929	0.000

At T3, the experimental group exceeded the control group on CA (3.740 vs. 3.653), *p* > 0.05 (Table 4), partial *η*^2^ = 0.003. At T3, the experimental group exceeded the control group on CDMSE (3.601 vs. 3.589), *p* > 0.05 (Table 4), partial *η*^2^ = 0.000.

Thus, H3 was supported at post-test (T2), but the between-group differences were no longer significant at follow-up (T3).

## 4 Discussion

### 4.1 Career adaptability outcomes

The findings provide strong support for Hypothesis 1, confirming the effectiveness of the structured Career Planning Education (CPE) intervention in enhancing Career Adaptability (CA) among the experimental group. Specifically, the significant increase in CA from pre-test to post-test, coupled with the experimental group’s significantly higher scores compared to the control group at post-test, highlights the immediate impact of the intervention.

However, a slight decline in CA scores was observed at follow-up (T3), with the experimental group scoring lower than at post-test. Although the Time 3 scores remained higher than the pre-test and were still above those of the control group, these differences were not statistically significant. This reduction may reflect the natural attenuation of intervention effects over time, particularly in the absence of continued reinforcement or structured follow-up activities. It is also possible that students, having returned to their regular academic routines, shifted focus away from career-related tasks, which could have contributed to the diminished gains. Given that the observed magnitudes at T3 were very small, the lack of statistical significance may also reflect limited statistical power at the current sample size rather than a true absence of effects.

In contrast, the control group did not exhibit significant changes across any of the three time points, suggesting that natural development or repeated questionnaire exposure alone was insufficient to meaningfully enhance CA. These results suggest that targeted career planning education can promote students’ adaptability in the short term in navigating career development tasks and transitions.

From a theoretical perspective, the CA findings are consistent with Career Construction Theory (Savickas, 2005), which conceptualizes adaptability as a set of resources for managing career tasks and transitions. In the present study, however, CCT was not used in isolation or in a simple one-to-one mapping onto the four CA dimensions. Rather, activities for allied medical students combined CCT with Social Cognitive Theory by using the same teaching tasks to build adaptability resources and to provide efficacy-enhancing experiences. For example, sessions on medical career paths asked students to draw career lifelines, construct family career trees and write “future-self” reflections linked to clinical roles, while discussing real cases of specialty choices. These exercises were intended to strengthen career concern, control and curiosity (CCT), and at the same time to offer mastery and vicarious experiences that support self-appraisal and information gathering in medical career decision-making (SCT). Group work in which students compared residency and specialty options, analyzed doctor-patient communication scenarios and planned their own stages of training was designed to enhance career confidence and openness to change, while providing structured practice in planning and problem solving. Taken together, these elements suggest that the CPE module operated as an integrated, theory-driven curriculum rather than a simple direct intervention targeting the four CA dimensions alone.

The findings of this study are consistent with prior research demonstrating the value of structured, theory-informed interventions in improving career adaptability (CA). For example, Teychenne et al. (2019) showed that even a brief online module, when aligned with Career Construction Theory, could produce measurable gains in CA among undergraduates. Similarly, Kepir Sávoly and Tuzgol Dost (2020) reported that a transition-focused program led to sustained CA improvements among senior students, echoing the short-term gains in the present study. Bal and Arikan (2020) further emphasized the effectiveness of multi-session curricula that incorporate self-reflection and decision-making, core elements also embedded in the current CPE intervention. In the context of medical education, Hur et al. (2018) found that targeted coaching reduced career indecision, a result that complements the improvements in career adaptability observed in our participants. Moreover, Gai et al. (2022) provided evidence from Chinese students that motivational interviewing can enhance CA, particularly among those with low baseline adaptability, aligning closely with the gains seen in our experimental group. More recent curriculum-integrated, online and life-design interventions show similar patterns of improvement in CA (Kim, 2022; Wetstone and Rice, 2023; Cardoso et al., 2022; Soares et al., 2022).

### 4.2 Changes in career decision-making self-efficacy

The results provide clear evidence that the Career Planning Education (CPE) intervention had a significant and positive impact on students’ career decision-making self-efficacy (CDMSE) in the short term. The substantial increase from pretest to posttest within the experimental group, as well as the significantly higher posttest scores compared to the control group, demonstrate the immediate effectiveness of the program in boosting confidence in making informed career decisions.

Although a slight decline in CDMSE was observed at the follow-up (T3), the experimental group still maintained a within-group score significantly higher than at pretest, indicating attenuation with limited maintenance relative to baseline; notably, between-group differences at T3 were nonsignificant. The lack of a statistically significant difference between the experimental and control groups at Time 3 may reflect the attenuation of intervention impact in the absence of ongoing reinforcement. It is also important to consider that the control group exhibited a relatively higher baseline score at pretest, which, although not statistically significant, may have reduced the observable between-group difference at follow-up. In addition to attenuation and baseline imbalance, because the observed effects at T3 were very small, our sample may not have had enough power to detect them.

In contrast, the control group showed no significant changes across the three time points, implying that the natural maturation process or repeated exposure to the measurement alone was insufficient to significantly enhance their decision-making self-efficacy. Taken together, these findings highlight the short-term efficacy and partial long-term sustainability of the CPE intervention, while also pointing to the importance of continuous support in maintaining growth in CDMSE.

From a theoretical standpoint, these CDMSE findings are consistent with Social Cognitive Theory (Bandura, 1977, 1986), which views self-efficacy as shaped by performance accomplishments, vicarious experiences, verbal persuasion and physiological states. For example, group tasks in which students compared specialty options, analyzed doctor-patient communication scenarios or discussed typical dilemmas faced after employ were designed to provide mastery experiences and modeling, as students practiced information gathering, goal selection, planning and problem solving in realistic medical contexts. Class presentations and peer feedback on individual career plans offered further opportunities for verbal persuasion and for observing how peers approached similar decisions, while the use of small, self-selected groups and structured reflection aimed to provide an emotionally safe environment for exploring uncertainties about the future. These same activities were also intended to strengthen adaptability resources, so that the module operated as an integrated CCT- and SCT-informed curriculum rather than two separate, direct interventions targeting CA and CDMSE in isolation.

The present findings align with existing studies demonstrating the effectiveness of theory-based career interventions in enhancing CDMSE. Similar improvements were reported by Talib et al. (2015) and Lam and Santos (2018), whose programs emphasized identity exploration and career knowledge. While those interventions yielded short-term gains, the current study extends the evidence by demonstrating partially sustained effects at follow-up. Studies by Osborn et al. (2020) and Chukwuedo et al. (2021) further support the value of structured guidance, reflection, and metacognitive skill-building in strengthening decision-related self-efficacy. Additionally, research by EI Naggar et al. (2021) and Putri et al. (2019) highlight the role of contextualized career support among medical students. Meta-analytic evidence also points to robust effects: Özlem (2019) found that career interventions for university students were associated with a large overall increase in CDMSE, and Sharapova et al.’s (2023) review of school-based career guidance reported consistent benefits for students’ career-related beliefs, including decision-making self-efficacy. Echoing our pattern, a recent review of school-based career interventions reported consistent gains in both CA and CDMSE, with stronger short-term effects on CDMSE and weaker maintenance at follow-up (Wang et al., 2024). This supports our finding of within-group maintenance for CDMSE at T3 and underscores the need for post-course boosters and spaced practice.

## 5 Practical implications

The slight decline observed at follow-up suggests that without ongoing reinforcement, students may struggle to maintain the same level of adaptive functioning. This highlights the need for longitudinal support mechanisms, such as personalized career counseling and periodic check-ins, to help students retain and apply their adaptability and decision-making skills over time.

Furthermore, the findings underscore the importance of broader institutional involvement. Career development should not be confined to elective courses or career centers alone. Faculty across disciplines, including those teaching major-specific or clinical subjects, should be encouraged to integrate career-oriented discussions into their instruction, reinforcing adaptive competencies across diverse academic contexts.

Finally, given the immediate gains and partial decline observed, institutions may consider offering structured career planning education on a recurring basis. An annual curriculum would allow students to revisit and deepen their self-awareness, respond to new developmental tasks, and sustain long-term growth in both adaptability and career decision-making self-efficacy.

## 6 Limitations

This study has several limitations. First, the sample was drawn from a single university in Jilin Province, which may introduce institutional bias and limit the generalizability of the findings. Future research should explore whether similar outcomes can be observed in other regions and cultural contexts.

Second, the follow-up interval was short (4 weeks). Such a brief window captures only near-term retention and is insufficient to evaluate durability of effects, delayed gains. As a result, it does not capture long-term career development trajectories or actual employment outcomes. Future longitudinal studies are needed to assess the sustained impact of career planning interventions over time.

Third, given that the non-significant follow-up effects were associated with very small observed magnitudes, these null results may reflect limited statistical power at the current sample size rather than the true absence of effects and should be examined with larger samples in future.

Finally, although the CPE module was theory-informed and produced significant gains in CA and CDMSE, this study focused on overall effectiveness and did not include process measures or mediation analyses. Thus, the mechanisms through which the intervention affected students’ outcomes could not be examined directly. Future studies should incorporate intervention-mechanism research such as mediation to clarify why and how the program works.

## 7 Conclusion

This study demonstrates that a structured, theory-based career planning education program can significantly improve students’ career adaptability and career decision-making self-efficacy, producing short-term gains that attenuated by follow-up. These findings highlight the value of integrating career education into undergraduate curricula and contribute to the literature on scalable, inclusive, and evidence-based career development.

## Data Availability

The raw data supporting the conclusions of this article will be made available by the authors without undue reservation.

## Footnotes

1
www.wjx.cn
